# Abdominal wall hernia and mental health: patients lived experiences and implications for patient care

**DOI:** 10.1007/s10029-022-02699-3

**Published:** 2022-10-25

**Authors:** O. A. M. Smith, M. Mierzwinski, J. McVey, P. Chitsabesan, S. Chintapatla

**Affiliations:** 1 York Abdominal Wall Unit, York and Scarborough Teaching Hospitals, Wigginton Road, Clifton, YO31 8HE York UK; 2 School of Science, Technology and Health, York St. John University, York, UK; 3 Department of Psychological Medicine, York and Scarborough Teaching Hospitals, Wigginton Road, Clifton, YO31 8HE York UK

**Keywords:** Abdominal wall hernia, Mental health, Quality of life, Lived experience, Patient care

## Abstract

**Background:**

Abdominal wall hernia (AWH) affects mental health and mental health questions are frequently included within Patient-Reported Outcome Measures (PROMS) for this patient population. However, these questions have not been informed by the subjective lived experiences of mental health in AWH patients. This study is the first to qualitatively examine how AWH affects patients’ mental health.

**Methods:**

Fifteen patients were interviewed from a purposive sample of AWH patients until no new themes emerged. Interviews explored patient thoughts and experiences of AWH and mental health. Data were examined using Interpretative Phenomenological Analysis (IPA).

**Results:**

Three key themes pertaining to mental health were identified: “psychological and emotional distress”, “identity disruption” and “coping mechanisms and support systems”.

**Conclusion:**

Our findings illustrate that AWH is a pathology that can have a significant detrimental impact on people’s mental health. This impact has implications for patient care and can be treated and managed through better psychological support. This support may positively affect AWH patient’s experience and outcomes in terms of quality of life. This paper provides recommendations for improved AWH patient care in regard to mental health.

**Supplementary Information:**

The online version contains supplementary material available at 10.1007/s10029-022-02699-3.

## Background

It is well known that there is a link between mental health and physical health [1]. When using the term mental health, we adopt the World Health Organization (WHO) definition “mental health is a state of mental well-being that enables people to cope with the stresses of life, realise their abilities, learn well and work well, and contribute to their community” [2]. Abdominal wall hernia (AWH) is a pathology that affects physical health, but little is known about how it affects mental health. Some research is suggestive of a relationship between AWH and mental health [3–6]. Despite this, mental health research in the AWH field remains sparse.

A recent systematic review has highlighted tools used in assessing Qulaity of Life (QoL) in patients with AWH [5] where only two tools were noted to comprise items that addressed mental health—the HerQLes and the PTSD PCL5 [5]. Our work identifies mental health as one of the five themes affecting patients’ QoL [7] and in our independent review of AWH-specific quality of life tools, we have found that two of the six specific AWH quality of life tools (the HerQLes and the AHQ) include mental health [8–10].

More detailed insights on how AWH affects mental health from patients’ perspectives are needed. To understand how AWH has affected a patient’s mental health, it is critical to explore their lived experiences. Patients' lived experience is best captured through adopting a qualitative approach [11, 12]. To our knowledge, there are no qualitative studies pertaining to mental health experiences of AWH patients. The aim of this study is to explore the lived experiences of AWH patients and how this has affected mental health.

## Methods

### Study design

This study applied phenomenology, a research methodology used to qualitatively explore human issues and emotionally laden subjects [14, 15]. Deep insights into how AWH affected patients’ mental health were gained using Interpretative Phenomenological Analysis (IPA) [11]. The first four authors have been trained in IPA. Further to this, data and subsequent findings were triangulated by two abdominal wall hernia surgeons to ensure degrees of consistency in interpretation.

### Ethics

This research received approval from the Hull York Medical School (HYMS), Integrated Research Approval System (IRAS) and Health Research Authority (HRA). It was conducted in accordance with the Declaration of Helsinki and has been reported according to COnsolidated criteria for REporting Qualitative (COREQ) guidelines. The study protocol, patient information leaflet, consent forms, topic guide and interview schedules were designed and subject to an iterative approval process. All patients provided written and verbal informed consent.

### Recruitment

The technique used frequently in qualitative research, *“the identification and selection of information-rich cases for the most effective use of limited resources”* was employed [16, 17]. Participants were recruited from the AWH clinic in the York Abdominal Wall Unit. As mental health is a multi-dimensional construct, there was a variation amongst participants as per criteria of AWH. The maximum variation purposive sampling technique was used and included patients of all elements of Ventral Hernia Working Group grades as variables, thus including those with previous cancers, previous wound infection, stoma, intestinal fistula, COPD, diabetes, smokers, and obesity [15]. A letter of invitation along with an information sheet pertaining to the study and interviews was arranged. A more detailed overview of the research process is provided in the in-depth report of various key indicators concerning AWH patients’ quality of life [13].

### Research method

Interviews have proven to be “the gold standard” of qualitative research [18] and “the most productive mode for producing narrative data” [19]. Semi-structured interviewing techniques were adopted to draw out the lived experience of a phenomenon, in this case how AWH affects patients’ mental health [20]. Adapted from Stumpfegger [21], a schedule and topic guide were used to ensure a systematic and rigorous interview process. The topic guide was designed by two gastrointestinal and two plastic surgical consultants who operate within the York Abdominal Wall Unit. This guide was largely based on HRQoL parameters identified in the literature but included one sub-section on mental health and body image.

### Data collection

Fifteen semi-structured interviews were conducted by author OS. Three interviews were face-to-face and the rest were completed via telephone due to the COVID-19 pandemic. Each interview lasted between 45 and 90 minutes. Steps were taken to ensure trustworthiness by asking open questions, clarity check of answers, using prompts and probes, as well as specific mental health-related questions. The interview was audio-recorded and transcribed verbatim by OS and a medical secretary independent of the research team. Pseudonyms were used to ensure anonymity and confidentiality.

### Data analysis

Data analysis was an iterative process until thematic saturation. Participant transcripts were analysed using IPA within NVivo v12 (https://www.qsinternational.com/nvivo/home). Transcripts were analysed line by line to identify similar patients’ views that could be grouped together into a certain theme. Emergent themes were discussed with two gastrointestinal surgeons and two plastic surgeons who specialised in CAWR as well as an independent academic qualitative researcher, who does not have a surgical background (MM). This allowed triangulation of the findings as well as plausibility of results.

## Results

Fifteen participants were interviewed (eight men and seven women) with an age range of 36–85 years (median = 65 years). Table 1 provides a summary of participant characteristics. Supplementary file provides the participant biographies, providing more context to the following patient narratives. Participants often self-identified as “a patient” in their responses therefore, to be clear and consistent, the term “patient” is adopted from here on in.

**Table 1:** Study participant demographics^a^

Participant name	Sex	Age	VHWG grade	Hernia height X width (CM)	NHS/Private	Smoker	Diabetic	Wound infection	Stoma	Cancer	Fistula	BMI	Socioeconomic class	Employed	Post OP/Pre OP	Telephone interview
Agnes	F	65	2	17 × 17	NHS	Ex-smoker	No	No	No	Yes	No	29.6	Middle	Yes	Pre-op	No
Betty	F	63	1	30 × 20	Private	Never	No	No	No	No	No	26.1	Upper	Retired	Pre-op	No
Charlotte	F	68	4	30 × 20 and 60 × 12	NHS	Ex-smoker	Yes	Yes	No	No	No	38.6	Middle	Retired	Pre-op	No
David	M	61	2	7 × 7 and 9 × 9	NHS	Never	Yes	No	No	No	No	31.2	Lower	Yes	Pre-op	Yes
Eric	M	78	1	12 × 30	NHS	Ex-smoker	No	No	No	No	No	29.9	Middle	Retired	Pre-op	Yes
Frank	M	75	4	27 × 26	NHS	Ex-smoker	No	Yes	No	Yes	Yes	25.8	Middle	Retired	Post-op	Yes
George	M	45	4	7 × 8 and 9 × 13 and 16 × 20	NHS	Ex-smoker	No	Yes	Yes	No	No	30.5	Lower	Yes	Pre-op	Yes
Harry	M	84	2	23 × 15	NHS	Ex-smoker	Yes	No	No	Yes	No	28.8	Upper	Yes	Post-op	Yes
Ian	M	58	4	17 × 21	NHS	Ex-smoker	No	Yes	No	Yes	No	30.2	Middle	Yes	Pre-op	Yes
Joan	F	75	3	22 × 18 and 19 × 17 and Parastomal	NHS	Never	No	No	Yes	Yes	No	26.3	Middle	Retired	Pre-op	Yes
Kevin	M	74	3	20 × 15 and 15 × 15	NHS	Ex-smoker	No	No	Yes	No	No	32.4	Middle	Retired	Post-op	Yes
Lisa	F	39	1	10 × 12	NHS	Never	No	No	No	No	No	29.2	Middle	Yes	Post-op	Yes
Marge	F	36	1	5 × 20	NHS	Never	No	No	No	No	No	20.4	Middle	Yes	Post-op	Yes
Norman	M	77	3	30 × 20	NHS	Ex-smoker	Yes	No	No	Yes	No	24.1	Middle	Retired	Post-op	Yes
Ophelia	F	44	1	2 × 3 and 4 × 5 and 2 × 3	NHS	Never	No	No	No	No	No	30.7	Middle	Yes	Post-op	Yes

^a^Names given to participants throughout the research are pseudonyms, ensuring anonymity

**Fig. 1:**
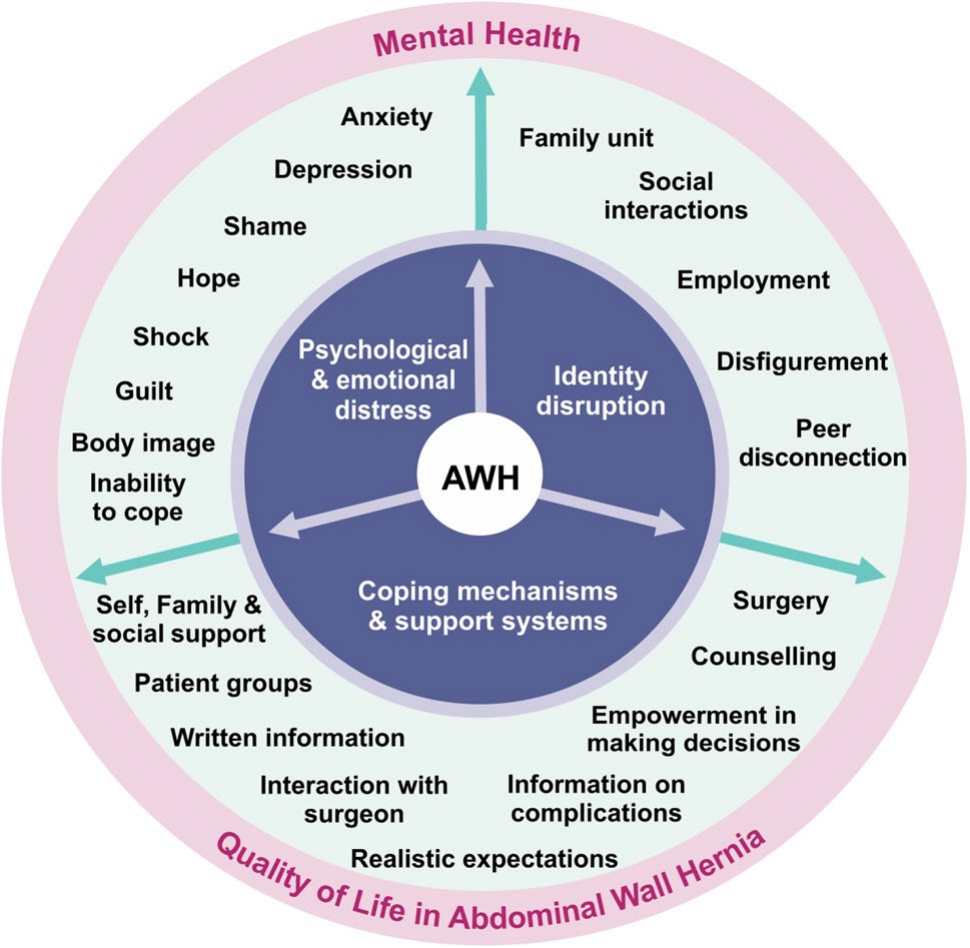
Mental Health themes relating to AWH patient’s Quality of Life

The results presented here are only the mental health aspects of QoL deemed important by AWH patients, with other themes reported elsewhere [13]. Three key themes pertaining to how AWH affected patients’ mental health were identified: “psychological and emotional distress”, “identity disruption” and “coping mechanisms and support systems”. Figure 1 explores these aspects from the patient perspective.

### Theme one: psychological and emotional distress

AWH detrimentally affected patients’ mental health, with patients describing regular episodes of anxiety, low mood and depression. Episodes were partly triggered by patients’ inability to perform previously enjoyed tasks and the psychological impact of a prolonged disease course.

*“The more aware of it (the hernia) and more self-conscious of it I was…then I suppose your confidence dips and…then anxiety increases as confidence dips, so you end up taking a step back from a situation rather than sort of being at the forefront of something.” (Ophelia)*

*“It has a corner of my life, because you do always have to consider what you’re doing as to what the effects of that are going to be…it impacts upon everything” (Charlotte)*

AWH restricted patients physically and psychologically, which heightened their degrees of self-consciousness and feelings of despondency during various stages of their hernia journey. As a result, many patients described adopting a risk averse approach to everyday tasks.

Risk aversity and episodes of anxiety could be triggered by patients’ awareness of possible complications that can occur during their wait to be optimised for theatre or postoperatively, as illustrated in this narrative.

*“I have been told about the risks of AWH by my surgeon…the pain increasing…the severity of it…and the possible consequences of leaving it alone, which are quite frightening now, because septicaemia, gangrene, strangulation of the bowel, already having had major bowel surgery, I really don’t want to lose another big part of bowel…I woke up in the night literally sweating with the pain…it was…shocking…because I had been warned of the consequences of a bowel rupture…I don’t want to be miles from anywhere (a hospital) if this is going to be the precursor of a rupture…it is just constantly at the back of my head…I’ve been more breathless, and again I’m wondering, because some of the injection (botox) was here, as it effected more areas than just the stomach area, or is it anxiety, or am I nauseous and breathless because I’m anxious” (Agnes)*

Given the possible detrimental consequences involved with AWH, patients’ fear was rational and prompted many to opt for operative management and surgery immediately after diagnosis. There was hope that surgery would be a solution to their problems. This hope left some patients not eligible for immediate surgery feeling disheartened and could fuel episodes of anxiety leading many of them to take a risk averse approach to everyday tasks pre and post operation.

Patients’ episodes of anxiety and feeling disheartened were also partly affected by concerns surrounding their body image.

*“Mentally it’s a vile thing.” (Eric)*

*“It obviously does have an effect, so it did, you know, it did make me feel, yeah, very low about you know my body image.” (Lisa)*

The appearance of AWH made patients feel anxious regarding certain activities in public, whilst heightening feelings of negative self-perception concerning their body image. Patients’ described periods of low moods, low self-esteem, and fears of being negatively judged by peers and were laden with varying levels of shame, disgust, embarrassment and occasionally guilt.

### Theme two: identity disruption

The psychological and emotional distress outlined above was partly impacted by, and contributed to, patients’ experience of identity disruption. Patients often described disruptions in their sense of self through their perceptions of changes in their role as a friend, family member and worker. Most patients cited how the psychological and emotional distress caused by AWH had negative social interactions, disrupting their friendship and family unit networks.

*“It (the hernia) was making me miserable. It was affecting me seeing people and going out. I would feel quite self-conscious, and it would take me ages to just get ready and choose what to wear, because of this constant reminder that what if somebody says I look pregnant today...I just never felt happy going out into a big social situation without feeling massively self-conscious about the way I looked…There were definitely occasions when I would feel fat and ugly even around my family and make up an excuse to not go.” (Ophelia)*

The disfigurement caused by AWH had heightened patients’ negative self-perception, leading them to withdraw from social gatherings in public, impacting partly on their role as friend.

Whilst frustrated by having to adopt avoidance tactics, patients’ levels of frustration were heightened further when describing the impact AWH had on their ability to perform desired and expected family roles. Their inability to perform previously taken for granted everyday practices disrupted their identity as a family member.

“*I stopped doing things with my grandchildren like I would normally do. One of my grandchildren is disabled and I enjoyed going out boating with him. But now I cannot do it and I feel terrible about it. But I just can’t. (Frank)*

*“Like if she wanted a cuddle (daughter) I would sit on the sofa without picking her up, you know and stuff like that. So I just adapted. But yeah, it wasn’t very nice.” (Marge)*

The disfigurement and/or reduction in physical ability caused by AWH affected how patients perceived their work and triggered self-doubts concerning their ability to successfully perform their role as a worker.

*“I’ve lost a lot of interest in work. Work is hard at the moment…I panic now going to work. I should maybe change jobs. I don’t know, I don’t know what to do.” (Charlotte)*

*“I got a job on a temporary rolling basis and that’s when the infection hit, but unfortunately lost that job because of it because they couldn’t afford to keep me on long time sick” (Ian)*

AWH had caused growing levels of physical disability which detrimentally impacted the ‘worker identity’ of those patients reliant upon certain degrees of physical function. Given the disruption that AWH had on patients’ various levels of identity, peer disconnection and reduced social interactions, it is understandable why patients often yearned for a return to their pre-AWH former self.

### Theme three: coping mechanisms and support systems

Patients yearning to return to their former pre-AWH selves impacted their coping mechanisms. To better manage their mental health, patients reported physical coping mechanisms.

*“I have a backpack and I put the shopping in my backpack, or I have a trolley if I really have to do a big shop” (Charlotte)*

*“I like to be in a shouting distance of home so that if anything did happen I could get back. I didn’t have overnight stays or anything like that.” (Frank)*

These physical coping mechanisms were largely self-driven and grounded in pragmatism, adaptation and risk adversity.

As well as individual coping mechanisms, the most frequently cited and significant were socially informed coping mechanisms. These included seeking support from peers and family and it is understandable these trusted family and friends formed a central part of patients’ coping mechanisms.

*“I’ve had to learn how to ask for help, and it’s quite a strange sort of thing to have to start doing, you know, to reprogram your mind. (Ian)*

*“My family and friends are really supportive, but they don’t fully understand how I feel.” (Ian)*

Alongside physical and social coping mechanisms, patients came to psychologically realise that the AWH journey was one of slow progressive change with multiple setbacks, distressing episodes and fluctuating emotions. One aspect of this journey was patients’ need to gain a greater awareness of what AWH is and what AWH surgery entails. Patients often used this information to inform their expectations concerning a return to their former pre-AWH self.

*“I didn’t realise how complicated it was (the surgery). At first, I just went with, sort of with the flow, thinking. My surgeon was really positive that it’s going to make, it will be better, and it will definitely be better for your back. So, I thought, oh, brilliant, that’s fine…I didn’t know like I was going to be on an epidural for like all them days” (Marge)*

*“I still see myself getting back to normal and I’m not sure quite how realistic that is, but that’s the million-dollar question…I think I’ve probably become more realistic about what I can achieve, because I used to set myself, right today I’m going to do this, this, this, this and this, and then if I didn’t, if I started to fall behind time, I’d start to get stressed.” (Betty)*

Patients felt that surgery was their only option to regain their former pre-AWH identity. However, some patients were ill-prepared in terms of their knowledge of their readiness to be operated on, the operative repair and postoperative complications. This knowledge gap contributed to some patients often holding unrealistic expectations in terms of outcomes concerning recovery time and aesthetics.

Part of the developing patients’ awareness of AWH involves pre-operative clinic sessions, which patients referred to as being informative, revealing and hard-hitting. Patients frequently cited positive surgical encounters and emphasised the important role surgeons played in influencing their AWH journey.

*“My surgeon seemed to understand when I said that I didn’t want the interruption in my life, I wanted to get on with living.” (Joan)*

Patients trusted the surgical team and appreciated their due diligence, empathy and understanding of the surgical necessity. However, some patients expressed the need for more information on possible complications, the length of recovery and desired more post-op check-up appointments.

*“It would have been nice just for another follow up check, I suppose, for reassurance more than anything. I had a 3 month follow up appointment. I would have liked another one.” (Joan)*

Patients’ reflections illustrate the levels of anxiety AWH had caused them. Interestingly, patients acknowledged that whilst they desired more knowledge, greater insight could make them more anxious. Further post-operative consultations would alleviate patients’ fears and anxieties by allowing them to seek greater reassurance on their recovery and progress.

The use of various coping mechanisms and engagement with different support systems alleviated some patients’ levels of anxiety and feelings of despondency; both proving enlightening and increasing their low level of self-esteem. However, it is important to note that even patients who actively engaged in coping mechanisms and support systems often reported psychological and emotional distress due to AWH.

## Discussion

The mental health themes identified in this paper were consistent across patients regardless of VHWG grade, hernia size, age or sex. The use of purposive sampling in qualitative research allowed for identification and selection of information-rich cases related to the phenomenon of interest and the use of maximum variation sampling captures all elements that go to make up VHWG grades. The consistent detrimental effects that AWH has on patients’ mental health stresses the importance of focussing on this topic. Literature suggests that surgeons disengage from patients with mental illness [22–25]. Key findings in this paper can illustrate the strong and complex relationship between physical, psychological and emotional distress caused by AWH.

Our study presents how patients perceived the relationship between AWH/ComplexAWH and their mental health. Mental health is a multi-dimensional concept that people experience differently and fluidly. In this study, patients tell us that their mental health is affected in some way shape or form throughout the AWH/CAWH journey, fluctuating depending on biases or patient interpretation. We use these degrees of variance affects to inform our critical engagement with HRQoL measures and implications for patient healthcare.

Patients anxiety, low mood and depression, alongside feelings of embarrassment, disgust and shame and a heightened sense of negative self-perception and negative self-worth often centred on how AWH affected their bodily aesthetic and their ability to perform everyday tasks [26]. These psychological and emotional outcomes left patients yearning for a return to their pre-AWH sense of self and were partly triggered by, and contributed to, patients’ frustrations at not being able to fully perform their role as friend, family member and/or worker. AWH disrupted aspects within patients’ previously solid identity, which patients interpreted as an unwanted part of their authentic self and body image. Patients appeared to derive a significant part of their identity from, and through, their physical body.

One key theme to emerge was patients’ referral to their ‘AWH journey’. Patients often described this journey using powerful metaphors, a trend also used by cancer patients [21, 27]. Furthermore, patients may arrive at clinic with premorbid poor states of mental health or negative body image due to other factors i.e. co-morbidities and obesity. Previous and current psychological and emotional traumas alongside bespoke coping experiences make patient care challenging. Some patients may have already tried different coping mechanisms with varied levels of success.

### Study limitations

Whilst key benefits of a qualitative study are evidenced above, this research approach can lead to some potential limitations due to inherent humanistic nature of qualitative research. We tried to manage potential limitations to ensure credibility and trustworthiness throughout the research process using steps outlined in methods section (i.e. sampling and triangulation) and disclosed in the supplementary files illustrate the degrees of credibility and trustworthiness undertaken [12]. In this study, most of the interviews had to be completed via telephone due to COVID-19. Some subtle nuances in patient body language may have been missed.

Any study concerning mental health effects would benefit from detailed patient psychiatric pathology before, during and after AWH/CAWH. This information would enable researchers to better ascertain causal or strong correlative coefficients and dismiss any extraneous variables. However, this research is not viable due to the unpredicted nature of AWH and the stages patients enter diagnosis and clinic, and the lack of continued access we have with patients several months post surgery.

### Development suggestions

To improve psychological support we, the authors, would recommend certain timely interventions -

Pre-referral

1. Information for patients about where to get help for depression and anxiety (www.nhs.uk/conditions/stress-anxiety-depression). All patients can refer themselves to Improving Access to Psychology Therapies (IAPT) programme (2008), a UK wide scheme to increase access to talking therapies.

At initial review

2. Acknowledgements and address this need on their first consultation. At York Abdominal Wall Unit, we use a health questionnaire where one section allows the patient to write down in their words how their mental health is and we participate in gathering information that allows shared decision making based on what is important to the patient and by addressing patient concerns.
3. We provide written information about AWH/ComplexAWH complications in a way that does not lead to overload and gives realistic expectation in terms of pre-operative optimisation.

At preparation

4. Pre-habilitation health care workers must include psychological strategies and allow time for patients to manage this information for shared decision making. Consider producing a leaflet on getting fitter for AWH surgery with advice on mental health including approaches and local resources to deal with mental health (our unit’s local information leaflet is shown in the hospital website Our Commitment to You (yorkhospitals.nhs.uk)
5. The development of education materials to help patients, preferably co-produced between staff and patients using Plain English and other languages. Patients with AWH have anxiety and reduced confidence over time and need tips on how to deal with comments from others. The Changing Faces website (https://www.changingfaces.org.uk) is a good model for this. To improve information and expectations regarding surgery, consider providing written information about AWH surgery and its complications and a leaflet on recovery physiotherapy after AWH surgery.

Pre- and post-surgery

6. A Holistic Needs Assessment (HNA) in the months leading up to surgery may be beneficial, in the same way it is used in cancer management. For a percentage of cases, especially those with no support networks/previous trauma, it would be desirable for the surgical team to have the ability to refer a patient for individual psychology sessions within the Acute Trust, pre or post-surgery.

## Conclusion

This study is the first to qualitatively examine how AWH affects patients’ mental health. Mental health is a multi-dimensional concept that people experience differently and fluidly. This paper provides rich, descriptive patients’ tales of living with and managing AWH. Central to patients lived experiences were recurring themes of physical, psychological and emotional distress. This distress and self opprobrium was caused by the advent of patients AWH and fuelled by how it had disrupted their identity. Various coping mechanisms and engagement in support systems somewhat alleviated patients’ levels of distress. Despite such mechanisms and systems, patients AWH journeys were often long, complex and non-linear. It was clear that AWH detrimentally affected patients’ mental health, had significant consequences and impacted various other aspects of their quality of life. Yet mental health is often overlooked as a QoL domain in AWH patients. The key findings presented in this paper raise awareness of this central domain in AWH patients’ quality of life.

Patient mental health concerns are often not adequately acknowledged, recognised, or prioritised by surgical teams and they can be inconsistently experienced by patients. Whilst this can be explained through time and resource pressures and the complexity within mental health, it has significant implications for AWH patient care. For instance, a patient’s ability to successfully change behaviours is crucial to managing AWH such as preoperative weight loss. This example illustrates the inherent relationship between physical and mental wellbeing, which is experienced pre/post operation. This relationship can be better catered for and managed with a more open, holistic, and patient-centred care approach.

## Supplementary Information

Below is the link to the electronic supplementary material.

Supplementary file1 (DOCX 39 KB)
