# Correction to: Abdominal wall hernia and mental health: patients lived experiences and implications for patient care

**DOI:** 10.1007/s10029-022-02723-6

**Published:** 2022-11-28

**Authors:** O. A. M. Smith, M. Mierzwinski, J. McVey, P. Chitsabesan, S. Chintapatla

**Affiliations:** 1 York Abdominal Wall Unit, York and Scarborough Teaching Hospitals, Wigginton Road, Clifton, YO31 8HE York UK; 2 School of Science, Technology and Health, York St. John University, York, UK; 3 Department of Psychological Medicine, York and Scarborough Teaching Hospitals, Wigginton Road, Clifton, YO31 8HE York UK

**Correction to: Hernia** 10.1007/s10029-022-02699-3

The article Abdominal wall hernia and mental health: patients lived experiences and implications for patient care, written by O. A. M. Smith, M. Mierzwinski, J. McVey, P. Chitsabesan and S. Chintapatla, was originally published electronically in SpringerLink on 25 October 2022 without open access. After publication online with the author(s)’ decision to opt for Open Choice the copyright of the article changed on 19 November 2022 to © The Author(s) 2022 and article is licensed under a Creative Commons Attribution 4.0 International License, which permits use, sharing, adaptation, distribution and reproduction in any medium or format, as long as you give appropriate credit to the original author(s) and the source, provide a link to the Creative Commons licence, and indicate if changes were made. The images or other third party material in this article are included in the article’s Creative Commons licence, unless indicated otherwise in a credit line to the material. If material is not included in the article’s Creative Commons licence and your intended use is not permitted by statutory regulation or exceeds the permitted use, you will need to obtain permission directly from the copyright holder. To view a copy of this licence, visit http://creativecommons.org/licenses/by/4.0/.

The original article has been corrected.

