# Supplementary material for: Abdominal wall hernia and mental health: patients lived experiences and implications for patient care
Source: Hernia. 2022 Oct 25;27(1):55–62. doi: 10.1007/s10029-022-02699-3 (PMC9595579; doi:10.1007/s10029-022-02699-3)
Supplement: Supplementary file 1 — Supplementary file1 (DOCX 39 KB) [file 10029_2022_2699_MOESM1_ESM.docx]

**Supplementary File 3:** Patient biographies

| Participant | Biography |
| --- | --- |
| Agnes | A 65 year old, retired woman. She has a good relationship with her husband and 3 adult children whom live far away. She has lived with her AWH for some years optimising for theatre by losing weight. During this time, she describes pain related to her hernia causing a severe impact on her life. She has experienced emotional difficulties that she believes are directly linked to her hernia for example significant anxiety related to the hernia, how it looks and the potential complications that may arise from it e.g. ischaemic bowel. She describes these as being so significant that she has adapted her behaviour and will not go on walks unaccompanied for fear of requiring urgent medical attention. She enjoys walking but trail difficulty and distances have been curtailed due to her hernia. |
| Betty | A 63 year old married woman who underwent private surgical care. She describes her hernia having a significant impact upon how she views herself and also, the distress of viewing herself as “pregnant” when in her sixties. It has impacted her mental health. Her first major experience with this was as a teenager when she suffered with anorexia. Thus, she feels her hernia has affected the progress she has made regarding her self-perception over time. She has found it very difficult to accept that she can no longer do the exercises that she previously enjoyed but, she has taken up various hobbies to help her such as sewing and designing loose garments to hide her hernia. She describes this as a “mental release” and it helps her to remain positive, ascribing the appearance of her hernia to feelings of low mood. She was taking antidepressant medication at the time of interview. |
| Charlotte | A 68 year old woman, who has a supportive partner. Since she has had her hernia, she has noticed a significant change in her body shape, which she finds distressing. This is so significant that she will no longer look at herself in the mirror. She describes giving up activities that she previously enjoyed like dancing because she finds this too upsetting. She acknowledges that although she has a strong relationship, the hernia has affected the intimate nature of the relationship with her partner due to pain. |
| David | A 61 year old man who has suffered with depression since the diagnosis of his hernia. He feels that his life has been completely restricted by his hernia to the point where it feels “like being in a prison cell”. Presently he is awaiting surgery, but this has been curtailed by the need to lower his BMI and minimise post-operative complications. He describes a vicious cycle where he wants to try to lose weight but then can’t owing to his depression and, that this is then followed by significant feelings of guilt. He has seen his GP about this issue and is now on antidepressants. He has grandchildren and feels that the hernia has affected his role in this capacity since he is apprehensive to lift them due to resulting hernia pain. He is married and states that he and his wife are unable to have intercourse owing to the hernia at present. |
| Eric | A 78 year old retired athlete. He was a caver, climber, fell runner, skier and mountain biker. He only recently stopped running an outdoor pursuit center and has a very strong identity as someone who is fit and active. He describes his hernia as having restricted his ability to pursue sport, which is a great love in his life. He is a field archer, which he describes as physically demanding, but has had to cut back on the walking and has had to adapt his stance due to the pressure it places on his abdomen. He is conscious of this around other participants because it looks “ungainly”. He and his wife have a supportive relationship, but he acknowledges that his hernia has significantly impacted their lives e.g. they no longer travel due to lack of insurance cover. It has also affected how he perceives himself to the point where he, too, will not look at himself in the mirror and his wife is not allowed to see him undressing or undressed. |
| Frank | A 75 year old man who has undergone CAWR. He describes his quality of life being much improved since he has had his operation and that he is now able to do a variety of activities that were previously curtailed by the hernia. When he did have his hernia, he described various adaptations that he made such as using a lady’s bicycle that is easier to mount (which he continues to use) and having to use women’s undergarments/spanx which were larger and more supportive although, equally embarrassing and emasculating. However, they decreased symptoms of discomfort related to his hernia. He described buying loose clothing to try to hide the hernia and felt distressed by looking like a “pregnant man”. |
| George | A 45 year old man employed in the trade industry. He is distressed by looking out of shape and having a pot belly. He struggles more with his stoma (for inflammatory bowel disease) since his hernia has increased in size - he describes more episodes of leaking and that, psychologically, he has never come to terms with it. It has affected his body image and his choice in clothing. It has had a significant impact on his work and personal finances. He describes losing a job and money due to sick days and, has even considered changing career but is reluctant to do so since it would result in decreased pay and he has fiscal commitments. This has had a resultant effect on his mental health, and he reports feeling low in mood to the point that his GP has now diagnosed him with depression. As time has gone on, he no longer engages in activities that he previously enjoyed either because 1. He physically can’t do it due to his hernia or 2. He no longer feels he has the energy or nature to do it because of his depression, which he believes is largely linked to the impact of his hernia on his life. He was taking antidepressant medication at the time of interview. |
| Harry | A 84 year old man who is post-op. He is an active gentleman who still works as an athletics coach despite his age. He describes being conscious of his looks and, again, described himself as a “pregnant man”. He has a stoma (cancer related) and described increased stoma leakage with increased hernia size as well as difficulty in fitting the stoma bags. Prior to having his operation he described that everything in his life required a lot of forward planning due to his hernia for example, knowing the location of toilets, stopping doing certain activities at work and no longer travelling long distances from home (which he used to do regularly for athletics meetings) in case something untoward happened related to his hernia. |
| Ian | A 58 year old grandfather. He has found it difficult to reconcile being unable to do the things he previously used to and finds it troubling to ask for help from others, to the point where he feels he has reprogrammed his mind. He described this as frustrating and that he just wants to get on with his life. Equally, he actively tries to remain positive by being involved with family. At the time of interview his son and daughter in law welcomed their first baby. He states that this has put things into perspective and swears that he will never waste a day again in his life again. |
| Joan | A 75 year old woman who has a parastomal hernia related to previous cancer. She is distressed by the self-perceived ugliness of her abdomen and, again, feels that she looks pregnant. She enjoys going to plays and dancing, but these activities have been curtailed. She has a supportive partner and describes their relationship as one of companionship rather than intimacy. Her children live far away, and she has little support from them. Over time she has become more accepting of her hernia and at present does not want an operation. She is an active member of hernia and stoma support groups which she finds helpful and an emotional outlet. |
| Kevin | A 74 year old retired, wheelchair bound man. Post-op. He was distressed by the fact his hernia became bigger and disguised it with specific clothing. Pre-operatively, he was frustrated that he could not engage with exercise programmes effectively owing to his mobility issues and felt that the hernia took over his life. He is heavily dependent on his supportive wife. |
| Lisa | A 39 year old PE teacher and mother of two children aged 5 and 2 years. She developed a noticeable AWH following her second child. Pre-operatively, her hernia affected her in a multitude of ways. She was unhappy about how she looked. This was made worse because previously she was incredibly fit and identified as an athlete. She was a ski instructor and personal trainer but had to give these up, resulting in financial implications. She was distressed by looking pregnant after having children. At work, she felt tremendous guilt when others needed to cover her PE classes or when she couldn’t demonstrate certain moves to her students. This reached a point when she considered giving up being a PE teacher and triggered feelings of low mood, although she was never formally diagnosed with depression. She described losing some of her identity as a mother since she struggled to pick up her children and was anxious about taking them out without her husband for fear that they would run off suddenly and that she would not be able to protect them. The intimacy of her relationship suffered. After the operation, she is far more confident in her appearance and can wear more fitted clothing and her working life has improved – she is now training as a yoga instructor. This has resulted in increased happiness that has permeated other aspects of her life. |
| Marge | A 36 year old married woman and mother of three young children. She described noticing a small lump after her third child and thought little of it until it became tender and increased in size. She was referred to the specialist hernia clinic whereupon she was very surprised to learn that she had an AWH, especially she is thin and physically fit. She works with prisoners and community offenders as well as a personal trainer in a local community center. She enjoys going for long runs and exercising with her husband and friends. Due to her hernia, she described feeling vulnerable around the prisoners particularly when they became angry since she knew she did not have the power in her core muscles. She feels that she has missed out in terms of exercising, which is the only thing she does for herself since much of her time is spent looking after her children or working. Her friends and family have been incredibly supportive during this time and post-operatively she has returned to doing the activities she used to enjoy, building endurance gradually. |
| Norman | A 77 year old retired man who lives with his wife. He described his hernia as ugly and enjoyed gardening immensely but struggled to do activities that involved lifting since in resulted in pain and discomfort. Post-op he denies any pain and can now fully mobilise about his garden with ease, engaging in activities he previously enjoyed. The has alleviated pressure that was once on his wife. |
| Ophelia | A 44 year old woman and mother of two – 8 and 5 years old. Prior to her operation she felt ugly and fat to the point where she would make up excuses not to go out with her husbands and her friends. She felt embarrassed about her profile and was asked on more than one occasion whether she was having another baby; an experience she described as mortifying. Such episodes triggered depression for which she received medication and her demeanor changed to be on the defensive. She suffered pain ascribed to her hernia particularly when bending or when her children pressed it. She adapted aspects of her life and states that doing things in this abnormal way became her normal and, that it was making her miserable. Since having her operation, she reports increased confidence and is even considering purchasing a bikini for this year’s family holiday. |

1. Bikhchandani J, Fitzgibbons RJ, Jr. Repair of giant ventral hernias. Adv Surg. 2013;47:1-27.

2. Fink C, Baumann P, Wente MN, Knebel P, Bruckner T, Ulrich A, et al. Incisional hernia rate 3 years after midline laparotomy. Br J Surg. 2014;101(2):51-4.

3. Halligan S, Parker SG, Plumb AA, Windsor ACJ. Imaging complex ventral hernias, their surgical repair, and their complications. Eur Radiol. 2018;28(8):3560-9.

4. Trujillo CN, Fowler A, Al-Temimi MH, Ali A, Johna S, Tessier D. Complex Ventral Hernias: A Review of Past to Present. Perm J. 2018;22:17-015.

5. van Ramshorst GH, Eker HH, Hop WC, Jeekel J, Lange JF. Impact of incisional hernia on health-related quality of life and body image: a prospective cohort study. Am J Surg. 2012;204(2):144-50.

6. Grove TN, Muirhead LJ, Parker SG, Brogden DRL, Mills SC, Kontovounisios C, et al. Measuring quality of life in patients with abdominal wall hernias: a systematic review of available tools. Hernia. 2020.

7. Lanier ST, Fligor JE, Miller KR, Dumanian GA. Reliable complex abdominal wall hernia repairs with a narrow, well-fixed retrorectus polypropylene mesh: A review of over 100 consecutive cases. Surgery. 2016;160(6):1508-16.

8. Smith O. A Phenomenological Study of the Lived Experiences of Patients with Complex Abdominal Wall Hernia (CAWH)

[M.D. Thesis]: Hull York Medical School, M.D. Thesis; 2021.

9. Krpata DM, Schmotzer BJ, Flocke S, Jin J, Blatnik JA, Ermlich B, et al. Design and initial implementation of HerQLes: a hernia-related quality-of-life survey to assess abdominal wall function. J Am Coll Surg. 2012;215(5):635-42.

10. Mauch JT, Enriquez FA, Shea JA, Barg FK, Rhemtulla IA, Broach RB, et al. The Abdominal Hernia-Q: Development, Psychometric Evaluation, and Prospective Testing. Ann Surg. 2020;271(5):949-57.

11. Smith JA, Flowers, P. & Larkin, M. Interpretative phenomenological analysis: Theory, method and research. London: Sage. 2009.

12. Noble H, Smith J. Issues of validity and reliability in qualitative research. Evid Based Nurs. 2015;18(2):34-5.

13. Smith OA, Mierzwinski MF, Chitsabesan P, Chintapatla S. Health-related quality of life in abdominal wall hernia: let’s ask patients what matters to them? Hernia. 2022.

14. Smith JA, Osborn M. Interpretative phenomenological analysis as a useful methodology for research on the lived experience of pain. Br J Pain. 2015;9(1):41-2.

15. Spichiger E. Family experiences of hospital end-of-life care in Switzerland: an interpretive phenomenological study. Int J Palliat Nurs. 2009;15(7):332-7.

16. Palinkas LA, Horwitz SM, Green CA, Wisdom JP, Duan N, Hoagwood K. Purposeful Sampling for Qualitative Data Collection and Analysis in Mixed Method Implementation Research. Adm Policy Ment Health. 2015;42(5):533-44.

17. MQ P. Qualitative research and evaluation methods. . Thousand Oaks, CA: 3rd Sage Publications;; 2002.

18. McCoyd JLM, Kerson TS. Conducting Intensive Interviews Using Email: A Serendipitous Comparative Opportunity. Qualitative Social Work. 2006;5(3):389-406.

19. Holt A. Using the telephone for narrative interviewing: a research note. Qualitative Research. 2010;10(1):113-21.

20. Starks H, Trinidad SB. Choose your method: a comparison of phenomenology, discourse analysis, and grounded theory. Qual Health Res. 2007;17(10):1372-80.

21. Stumpfegger E. Phenomenological Approach: From Research Philosophy to Research Design. 2015. p. 49-74.

22. Morgan JF, Killoughery M. Hospital doctors' management of psychological problems–Mayou & Smith revisited. The British Journal of Psychiatry. 2003;182(2):153-7.

23. McBride KE, Solomon MJ, Steffens D, Bannon PG, Glozier N. Mental illness and surgery: do we care? ANZ journal of surgery. 2019;89(6):630-1.

24. Chadda R. Psychiatry in non-psychiatric setting--a comparative study of physicians and surgeons. Journal of the Indian Medical Association. 2001;99(1):24, 6-7, 62.

25. Knaak S, Patten S, Ungar T. Mental illness stigma as a quality-of-care problem. Lancet Psychiatry. 2015;2(10):863-4.

26. Arora M, Barquera S, Farpour Lambert NJ, Hassell T, Heymsfield SB, Oldfield B, et al. Stigma and obesity: the crux of the matter. Lancet Public Health. 2019;4(11):e549-e50.

27. Hommerberg C, Gustafsson AW, Sandgren A. Battle, Journey, Imprisonment and Burden: patterns of metaphor use in blogs about living with advanced cancer. BMC Palliative Care. 2020;19(1):59.
